# The Application Value of the Renal Region of Interest Corrected by Computed Tomography in Single-Kidney Glomerular Filtration Rate for the Evaluation of Patients With Moderate or Severe Hydronephrosis

**DOI:** 10.3389/fphys.2022.861895

**Published:** 2022-05-09

**Authors:** Haixia Yin, Wenli Liang, Deshan Zhao

**Affiliations:** Department of Nuclear Medicine, The Second Hospital of Shanxi Medical University, Taiyuan, China

**Keywords:** glomerular filtration rate, hydronephrosis, dynamic renal scintigraphy, computed tomography, renal region of interest

## Abstract

**Objective:** This study aimed to investigate the application value of the renal region of interest (ROI) corrected by computed tomography (CT) in single-kidney glomerular filtration rate (GFR) in patients with hydronephrosis.

**Methods:** A total of 46 patients with hydronephrosis were divided into four groups based on their degree of unilateral hydronephrosis: a normal group (left kidney and right kidney) and three abnormal groups (mild, moderate, and severe hydronephrosis). GFR was measured using the two-sample method (tGFR). The single-kidney GFR of each patient was derived from differential renal function values in dynamic renal imaging multiplied by GFR. The single-kidney GFRs, including GFR from the Gates method (gGFR_single_) and CT area-corrected GFR (aGFR_single_), were compared with tGFR_single_. A paired-sample *t*-test and Pearson’s test were used for data analysis. *p* < 0.05 was considered statistically significant.

**Results:** There were no significant differences between aGFR_single_ and tGFR_single_ in patients in the normal, mild hydronephrosis, and moderate hydronephrosis groups (*t* = –0.604∼1.982, all *p* > 0.05), but there was a significant difference between them in the severe hydronephrosis group (*t* = 2.302, *p* < 0.05). There were no significant differences between gGFR_single_ and tGFR_single_ in the normal and mild hydronephrosis groups (*t* = 0.194∼0.962, all *p* > 0.05), but there was a significant difference between them in the moderate and severe hydronephrosis groups (*t* = 3.321, 3.494, *p* < 0.05). Both gGFR_single_ and aGFR_single_ were correlated with tGFR_single_, with aGFR_single_ being more strongly correlated (*r* = 0.890, *p* < 0.001).

**Conclusion:** In patients with moderate hydronephrosis, aGFR_single_ is more strongly correlated with tGFR_single_ than gGFR_single_. However, in patients with severe hydronephrosis and accompanying renal morphological changes, the aGFR_single_ measured by the renal ROI area-correction method using CT has higher accuracy and better clinical application value than the conventional gGFR_single_.

## 1 Introduction

Hydronephrosis is a common disease of the urinary system that is most commonly found in upper urinary tract obstructions (Klahr and Morrissey, 2002). Tc-99m-labeled diethylenetriamine pentaacetic acid (Tc-99m DTPA) dynamic renal imaging, which has been promoted as an effective method for evaluating renal function (Klahr and Morrissey, 2002), not only identifies simple renal pelvis dilatation and mechanical obstruction but also quantitatively analyzes renal function, especially separate renal function. The method introduced by Gates is commonly used in routine clinical settings; this method requires that the region of interest (ROI) of the kidney be delineated by the boundary of the kidney’s external shadow or the expansion of one or two pixels. If the kidney itself, or other external factors, lead to inaccurate delineation, the glomerular filtration rate (GFR) deviates from its true value. Due to the abnormal expansion of the renal pelvis and/or renal calyces in patients with moderate to severe hydronephrosis, the morphology of the involved kidney changes: the thickness of the cortex is thinner than that of a normal kidney, and renographic findings show a radioactive defect or slightly higher radioactivity in the involved kidney than in the background area. In this case, the delineation of renal ROI is prone to deviation. Several other factors that can disturb GFR values can also be measured using the Gates method. Furthermore, some studies have shown that renal depth correction using integrated computed tomography (CT) may have great significance for the accuracy of GFR determination except in the cases of moderate or severe hydronephrosis.

The two-sample method is considered to be accurate in the determination of GFR and serves as the standard method in the present study. The aim of this study is to compare the accuracy of the two-sample method with that of two different methods for the evaluation of single-kidney GFR (GFR_single_)—the Gates GFR_single_ (gGFR_single_) and CT area-correction GFR_single_ (aGFR_single_)—to identify which method is more accurate in the evaluation of GFR_single_ in patients with moderate or severe hydronephrosis.

## 2 Materials and Methods

### 2.1 Patients

A prospective study was conducted to analyze patients with hydronephrosis diagnosed by ultrasound. All patients underwent diuretic renography and integrated CT scans within 1 year. The study included 46 patients (20 male and 26 female), with an average age of 45.67 ± 13.52 years. There were 32 cases of unilateral hydronephrosis and 14 cases of bilateral hydronephrosis, with a total of 92 kidneys (32 normal kidneys and 60 kidneys with hydronephrosis).

Inclusion criteria: 1) the presence of unilateral or bilateral hydronephrosis and a referral for both Tc-99m DTPA dynamic imaging and renal homologous by a clinician or ultrasound physician; 2) GFR was calculated using the double-plasma method; 3) no contraindications to the administration of Tc-99m DTPA.

Exclusion criteria: 1) surgical history involving the urinary system, such as renal transplantation or drainage of hydronephrosis; 2) significant anomaly of the urinary system, including horseshoe kidney, solitary kidney, polycystic kidney, and renal tumors; 3) other risk factors, such as hypertension, severe cardiac insufficiency, or diabetes.

Written informed consent was obtained from all participants, and the study was conducted in accordance with the principles of the Helsinki Declaration.

### 2.2 Grouping

The enrolled patients were divided into two groups (normal and abnormal) according to the degree of unilateral hydronephrosis identified by CT. The severity of hydronephrosis was then determined according to CT imaging. Mild renal hydronephrosis was characterized by the renal parenchyma thickness not changing significantly; moderate hydronephrosis was identified by obvious renal calyceal hydronephrosis and dilatation, the disappearance of the cup mouth, an increase in renal volume, and part of the renal parenchyma becoming thinner; severe renal hydronephrosis was characterized by an enlarged renal outline, renal sinus area cystic dilatation and hydronephrosis, and a thinned or nearly atrophied renal parenchyma.

The normal kidney group consisted of 32 kidneys, including 19 left kidneys and 13 right kidneys. The abnormal kidney group consisted of 60 kidneys, including 28 left kidneys and 32 right kidneys. The abnormal group was further divided into three groups: a mild hydronephrosis group, a moderate hydronephrosis group, and a severe hydronephrosis group.

### 2.3 Diuretic Dynamic Renal Imaging

In all patients, 185MBq Tc-99m DTPA (Atom High-Tech Co. Ltd., Beijing, China), with a volume less than 1 ml and a radiochemical purity greater than 95%, was administered via bolus injection into the cubital vein. All patients were hydrated with 5–7 ml/kg of water 30 min before the renal scan and their bladders were emptied. The procedure was carried out using routine Tc-99m DTPA dynamic renal imaging with a dual-head gamma camera (SPECT, Discovery NM/CT 670; General Electric Company, New York, United States). With the patient in a supine position, the dynamic acquisition was performed using a gamma camera equipped with a high-resolution, low-energy collimator at the same time as a bolus intravenous injection of Tc-99m DTPA was administered into the patient’s forearm. Regions of the kidneys and bladder were placed in the center view of the gamma camera. Data were collected at 2-second intervals for 1 min and at 30-second intervals for 30 min, and 40 mg furosemide was administered intravenously at 20 min of image acquisition. After dynamic renal imaging was complete, integrated CT with 16 rows in a hybrid SPECT/CT was used to perform a low-dose scan (100 mA, 90 kV) on the bilateral renal areas, with the patient remaining in a supine position. Images with 5-mm thick transverse sections at 5-mm intervals were used for analysis.

### 2.4 Unilateral Renal Glomerular Filtration Rate Measurement

#### 2.4.1 Two-Sample Method

The International Scientific Committee of Radionuclides in Nephrourology recommended the two-sample method as a reference standard for the evaluation of GFR in clinical research (Maioli et al., 2020) with the two samples being taken from the vein in the patient’s forearm at 120 and 240 min, respectively, being found to be the most accurate. In the present study, two 5-ml blood samples were drawn at 120 and 240 min from the opposite forearm to the one in which the Tc-99m DTPA injection was administered. In accordance with the procedure for blood sampling, each sample was processed immediately after withdrawal.

The filtration counts of the two blood samples were tested and recorded on the basis of the procedure for ultrafiltration. The two-sample GFR (tGFR) of each patient was obtained from the data using the equation of the two-sample method, before being standardized using the patient’s body surface area. The tGFR was considered the standard GFR value for the patients in this study. The single-kidney tGFR (tGFR_single_) was calculated based on the differential renal function of two kidneys obtained by routine diuretic dynamic renal imaging.

#### 2.4.2 The Gates Method

Imaging data during the 2–3 min after radiotracer injection were used to calculate the GFR using the dedicated data-processing workstation linked to the gamma camera. The ROIs of the kidneys, backgrounds, and aorta were drawn by manual operation. After entering the patient’s weight and height, the gGFR_single_ was automatically calculated using the Gates algorithm.

#### 2.4.3 Renal Region of Interest Area Correction by Computed Tomography

**FIGURE 1:**
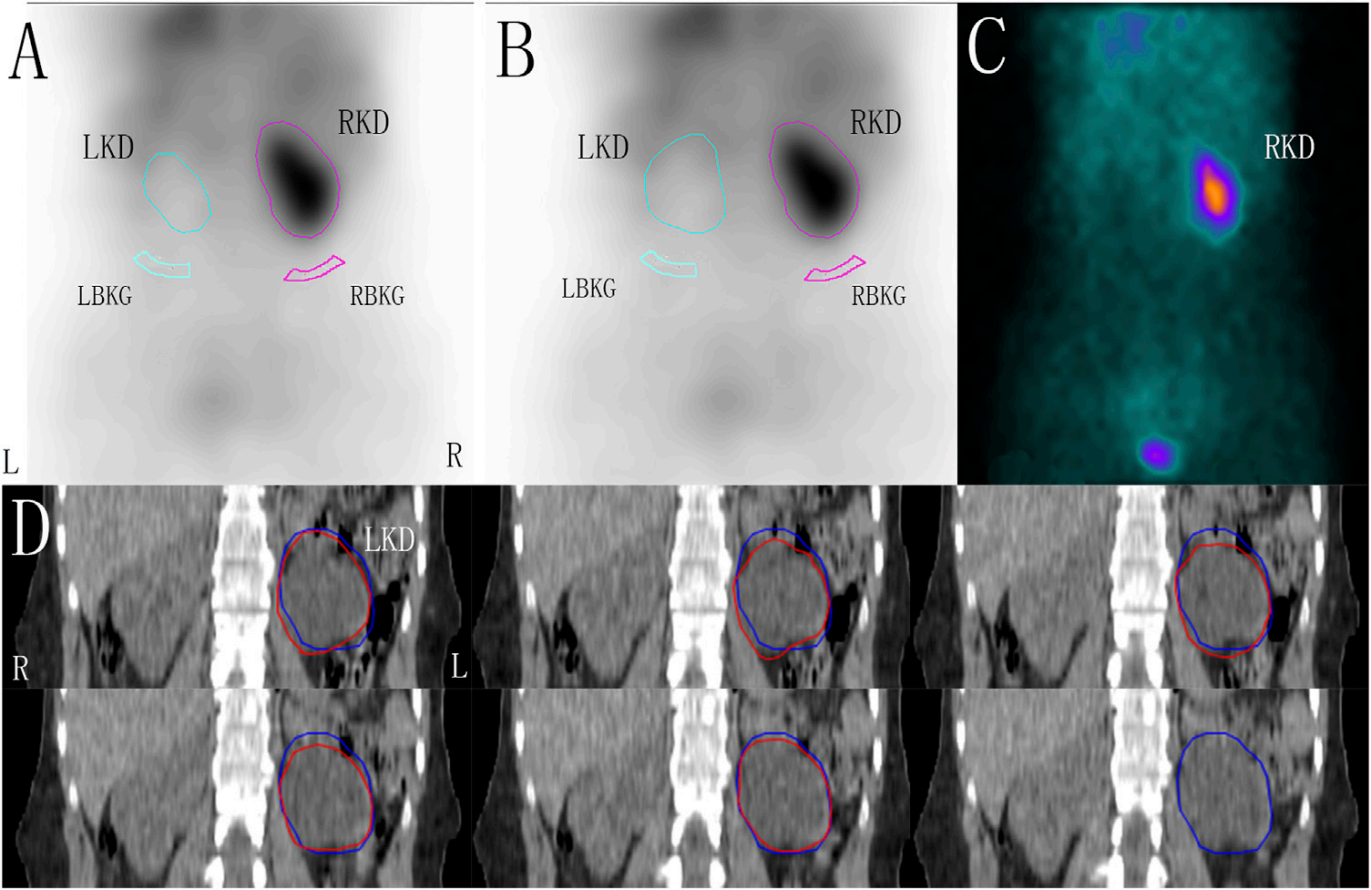
The renal region of interest (ROI) was corrected by computer tomography (CT) in a patient with left hydronephrosis, zoom: 1.5. **(A)** ROI of the kidneys and the background drawn using the Gates method. **(B)** Based on the maximum renal ROI area corrected by CT, the ROI of the kidneys and the backgrounds was drawn using the Gates method. **(C)** One-minute frames: shows the frame-per-minute image overlaid by the kidneys, cortex, background, and bladder. **(D)** ROIs of the left kidney were delineated layer by layer on CT coronal sectional images (red line), and the maximum ROI area of the kidney were redrawn and used as the renal ROI area from CT (blue line). LKD, left kidney; RKD, right kidney; LBKG, left background; RBKG, right background.

The ROIs of the kidneys were delineated layer by layer on CT coronal sectional images. After all unilateral renal ROIs were superimposed, the maximum ROI area of the kidney was redrawn and used as the renal ROI area from CT. According to the maximum renal ROI area and its contour from CT, bilateral renal ROIs were delineated on the renal images. The aGFR_single_ was then determined using the Gates method (Figure 1).

### 2.5 Statistical Analysis

Data were expressed as the mean ± standard deviation (SD). The GFR_single_ measurements obtained using the three methods were compared. Paired-sample *t*-tests and Pearson’s correlation analysis were used for statistical analysis. *p* < 0.05 was considered statistically significant.

## 3 Results

A total of 46 patients with 92 kidneys were included in this study. The gGFR_single_, aGFR_single_, and tGFR_single_ were normally distributed according to the results of the Shapiro–Wilk normality test.

### 3.1 Paired-Sample *T*-Test

**FIGURE 2:**
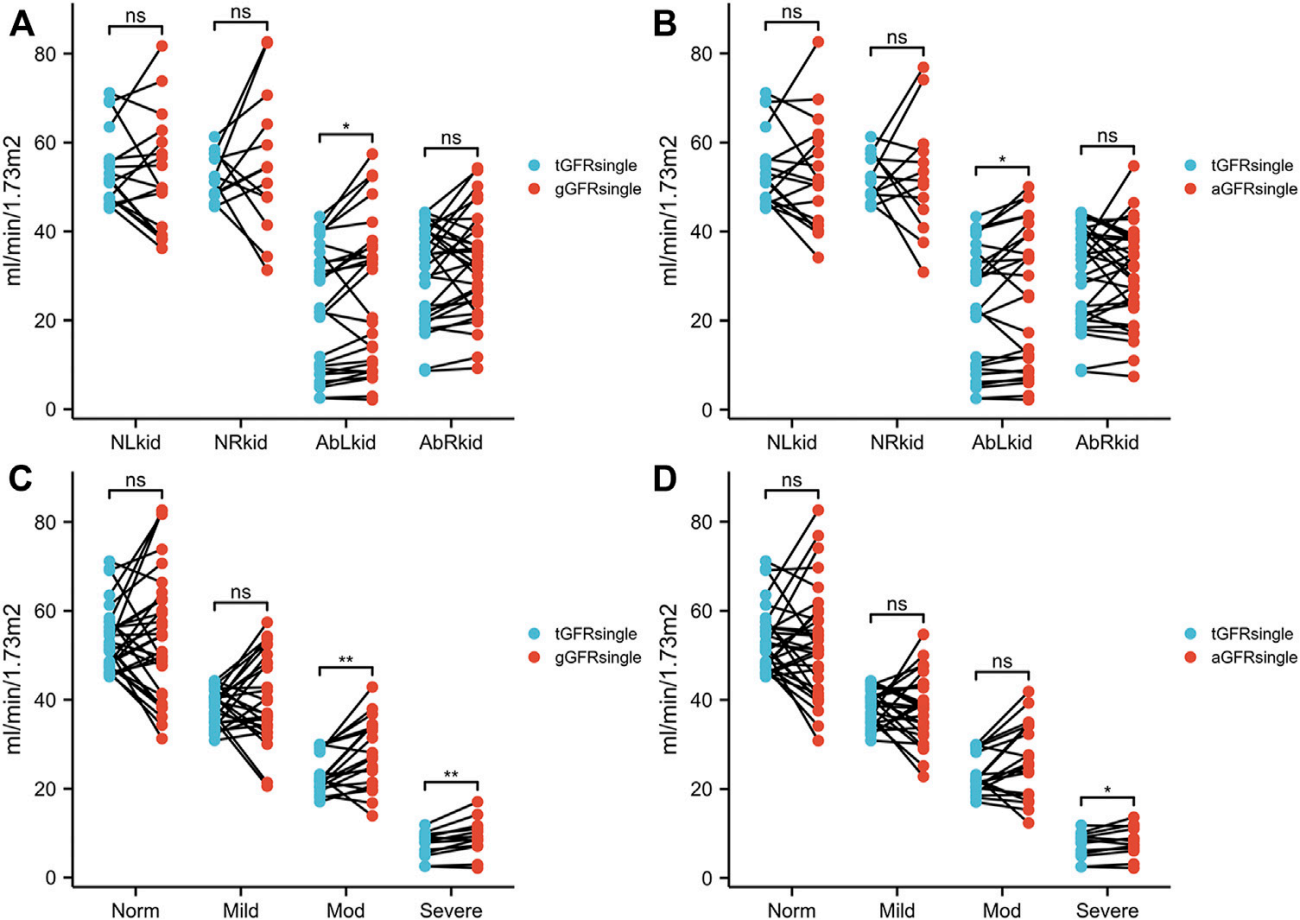
Comparison between gGFR_single_ and tGFR_single_ **(A, C)** and between aGFR_single_ and tGFR_single_ **(B, D)** in patients with hydronephrosis. NLkid, normal left kidney; NRkid, normal right kidney; AbLkid, abnormal left kidney; AbRkid, abnormal right kidney; Norm, normal; Mod, moderate; GFR, glomerular filtration rate; gGFR_single_, single GFR measured using the Gates method; aGFR_single_, single GFR measured by the renal region of interest area correction by CT method; tGFR_single_, single GFR, total GFR measured by the two-sample method multiplied by the differential renal function. *p* < 0.05 was considered statistically significant. ns, *p* ≥ 0.05, *p < 0.05, **p < 0.01.

There were no significant differences between aGFR_single_ and tGFR_single_ in patients in the normal, mild hydronephrosis, and moderate hydronephrosis groups (*t* = 0.653, 0.058, −0.604, −0.096, 1.982, all *p* > 0.05). In patients with severe hydronephrosis, however, the difference between aGFR_single_ and tGFR_single_ was statistically significant (*t* = 2.302, *p* < 0.05) (Figure 2; Table 1).

**TABLE 1:** Glomerular filtration rate (GFR) measured by three methods in the patients with hydronephrosis.

degree methods		Numbers of kidney	Two-sample method	Gates’ method		ROI area correction method by CT	
			tGFR_single_	gGFR_single_	T(p)	aGFR_single_	T(p)
Normal	L Kid	19	54.17 ± 8.37	52.90 ± 13.00	0.565 (0.579)	52.77 ± 12.25	0.653 (0.522)
	R Kid	13	52.60 ± 5.14	55.50 ± 16.23	0.681 (0.509)	52.39 ± 13.12	0.058 (0.954)
Abnormal	L Kid	28	22.37 ± 13.86	25.55 ± 16.66	−2.580 (0.016)^*^	24.60 ± 15.99	−2.136 (0.042)^*^
	R Kid	32	30.36 ± 10.61	32.43 ± 11.57	1.511 (0.141)	30.38 ± 11.85	−0.007 (0.994)
	Normal	32	53.53 ± 7.18	53.95 ± 14.20	0.194 (0.847)	52.39 ± 12.30	−0.604 (0.550)
	mild	27	38.38 ± 3.95	40.10 ± 9.78	0.962 (0.345)	38.24 ± 7.68	−0.096 (0.924)
	moderate	20	23.30 ± 4.37	27.62 ± 7.77	3.321 (0.004)^*^	26.09 ± 8.63	1.982 (0.062)
	severe	13	7.39 ± 2.88	9.08 ± 4.04	3.494 (0.004)^*^	8.24 ± 3.34	2.302 (0.040)^*^

L Kid, left kidney; R Kid, right kidney; GFR, glomerular filtration rate; gGFR_single_, the single GFR measured by Gates’ method; aGFR_single_, the single GFR measured by renal ROI area correction method by CT; tGFR_single_, the single GFR=Total GFR measured by two-sample method multiply by the differential renal function. *p* < 0.05 was considered significant statistically. *p < 0.05.

There were no significant differences between gGFR_single_ and tGFR_single_ in patients in the normal and mild hydronephrosis groups (*t* = 0.565, 0.681, 0.194, 0.962, all *p* > 0.05). In patients with moderate or severe hydronephrosis, however, the differences between gGFR_single_ and tGFR_single_ were statistically significant (*t* = 3.321, 3.494, *p* < 0.05) (Figure 2; Table 1).

### 3.2 The Correlation Between aGFR_single_, gGFR_single_, and tGFR_single_

**FIGURE 3:**
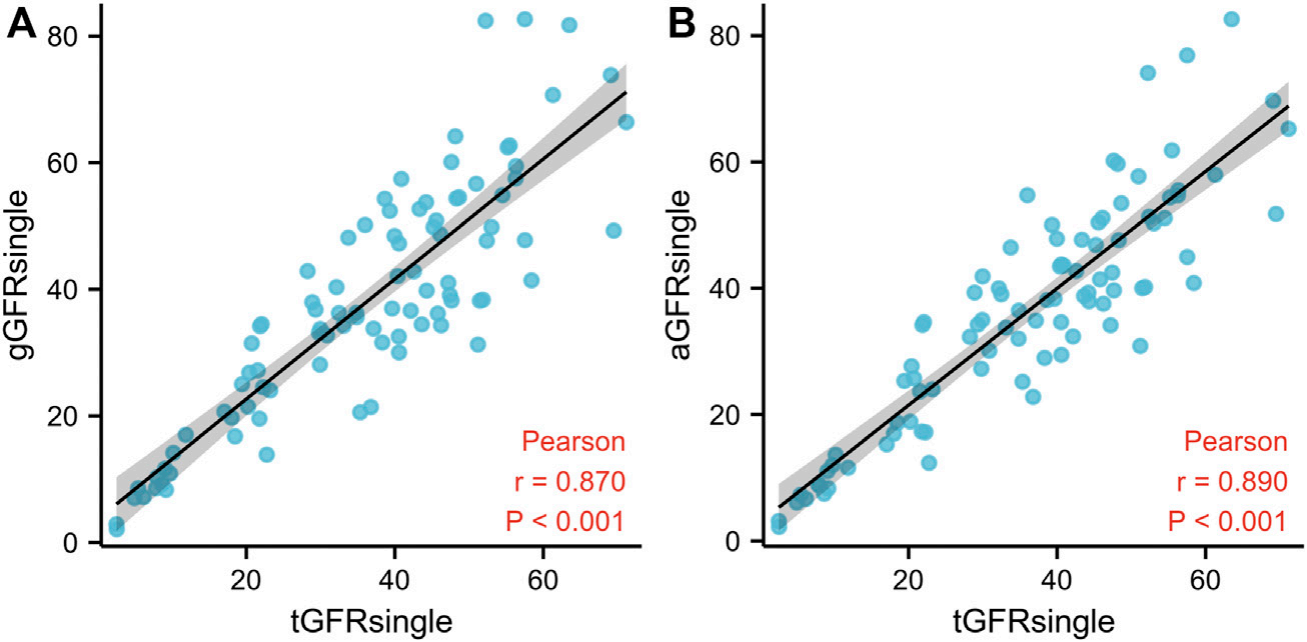
The relationship between gGFR_single_ and tGFR_single_ **(A)** and between aGFR_single_ and tGFR_single_ **(B)** in patients with hydronephrosis. The solid line represents linear correlation. GFR, glomerular filtration rate; gGFR_single_, single GFR measured using the Gates method; aGFR_single_, single GFR measured by the renal region of interest area correction by CT method; tGFR_single_, the single GFR, total GFR measured by the two-sample method multiplied by the differential renal function.

A good correlation (*p* < 0.001) was identified between aGFR_single_ and tGFR_single_ and between gGFR_single_ and tGFR_single_, with the former having the stronger correlation. The correlation coefficient between aGFR_single_ and tGFR_single_ was 0.890 (*p* < 0.001) (Figure 3).

## 4 Discussion

Hydronephrosis refers to the retention of urine in the dilated pelvis and calyces caused by obstruction, causing an increase in hydrostatic pressure and resulting in increased glomerular pressure and renal cortex atrophy, which ultimately diminishes GFR (Blaufox et al., 1996). Note that GFR is an objective index reflecting glomerular filtration function (Thotakura and Anjum, 2021) and is used as the main basis for assessing renal function (Stevens and Levey, 2009; Berg, 2006). It can be used to explain any abnormalities in the signs and symptoms of patients, as well as in the laboratory examination results of renal diseases (Thomas and Huber, 2006). It is of great significance for the diagnosis, the evaluation of the severity, development, and prognosis, and the selection of treatment options in renal diseases.

Using the two-sample method to measure GFR has been found to have better accuracy and higher clinical application value; however, it cannot identify the separate renal functions. Due to its good repeatability and relatively high accuracy, GFR measured by the Gates method is recommended for the clinical evaluation of total and separate renal function (Levey et al., 2020), but it is susceptible to many factors (Levey et al., 2007; Li et al., 2016). It also lacks accuracy in the GFR measurement of patients with severe hydronephrosis, so it is more suitable for patients with mild to moderate hydronephrosis. The results of the present study indicate that the Gates method is suitable for patients with normal kidneys or mild hydronephrosis.

The precise delineation of renal ROI determines the accuracy of GFR (Wei et al., 2020). Because of the increased hydrostatic pressure of the dilated renal pelvis and calycles in patients with moderate to severe hydronephrosis, the involved renal cortexes became thinner or even disappeared, and their clearance rate decreased. Initially, the distribution of the radiopharmaceutical was significantly reduced and/or defective in the involved renal cortexes in dynamic renal imaging, and the involved kidney in the following images was gradually seen or not clearly seen initially. At this point, errors in the renal ROI delineation were caused by the obscure border of the kidney during the 2–3 min after radiotracer injection, affecting the accuracy of GFR measurement. In addition, when identifying the involved renal ROI in patients with moderate and severe hydronephrosis, the boundary between the dilated collecting system and renal parenchyma was often unclear, which may have led to incorrect renal ROIs and false GFR values.

Troell et al. (Ma et al., 2019) found that the renal parenchyma area, which can be calculated by ultrasound, is highly correlated with GFR. Therefore, the method of drawing the renal ROI layer by layer on CT images and drawing the bilateral renal ROI on dynamic renal imaging according to the size and outline of the kidneys can outline ROIs more accurately, thereby ensuring they are not too large or too small. Due to the abnormal increase of involved renal size in patients with moderate to severe hydronephrosis, however, bilateral kidneys may overlap with the liver and the spleen, which are then easily included in the involved renal ROI delineated using the Gates method and can increase the radioactive counts of the kidneys, leading to an overestimation of GFR values. The present study found that the aGFRsingle of the normal right kidney was closer to tGFR than to gGFR. However, this difference was not found in the normal left kidney, which might be related to the fact that the right kidney is closer to the liver. When the right renal ROI was drawn using the Gates method, the delineated ROI was affected by the liver. In the abnormal groups, there were no significant differences between the GFR_single_ measured by the Gates method or the renal ROI area correction by CT and tGFR_single_ in the right kidney, whereas the opposite was true in the left kidney, which may be due to the fact that only 2 of the 32 abnormal right kidneys had severe hydronephrosis, compared with 11 of the 28 left kidneys. Due to the abnormal increase in the left kidney’s renal size in patients with severe hydronephrosis, its position was higher than that of a normal left kidney. When left renal ROIs were drawn using the Gates method, the delineated ROI often overlapped with the spleen and was therefore affected by it. In these cases, the aGFR_single_ was closer to the tGFR_single_ than the gGFR_single_.

Kidney depth is also an important factor affecting the measurement of GFR in the Gates method (Troell et al., 1984). It affects the vertical distance between the kidneys and the back surface of the body, resulting in a difference in γ-ray attenuation through soft tissue and affecting the net count rate of the two kidneys, thereby affecting the accuracy of gGFR. There are several methods by which renal depth can be measured, including ultrasound, CT (Gates, 1982), and lateral Tc-99m DTPA scintigraphy. Renal depth correction by CT is more accurate than by ultrasound. However, some studies have suggested that, while the correction of renal depth improves the accuracy of GFR measurement for patients in stages 1–2 kidney disease according to the Kidney Disease Outcomes Quality Initiative Guidelines, GFR measurement has low accuracy for patients in stages 3–5, and renal depth correction does not improve it. Therefore, the influence of renal depth on GFR measurement requires further study.

The present study had some limitations. Due to the small number of cases, there may be some bias in the statistical results, which can reduce the accuracy of the conclusions; therefore, it is necessary to increase the number of cases in subsequent research. Furthermore, the renal ROI delineated on the coronal plane of the CT images could not be automatically copied and pasted to the renal dynamic images, which limited the application of this method; therefore, the research and development of the image processing program are vital to promoting this technical method.

## 5 Conclusion

The GFR values of the patients with normal kidneys and most patients with mild hydronephrosis can be measured using the Gates method, and the involved renal ROI in this method does not need to be corrected. However, in patients with severe hydronephrosis, the conventional delineation of the involved renal ROI is prone to correction, and the patients’ therapeutic regimen needs a more accurate evaluation of the involved renal function and changes in kidney morphology. The ROI area correction using CT can obtain more accurate GFR values and can more accurately meet clinical needs. Therefore, if necessary, integrated CT scans should be used to correct the involved renal ROI for GFR measurement in patients with severe hydronephrosis, and this can be used as an effective supplementary or auxiliary diagnostic method in place of the Gates method (Inoue et al., 2016).

## Data Availability

The original contributions presented in the study are included in the article/Supplementary Material, further inquiries can be directed to the corresponding author.
